# A Case of New Delhi Metallo-ß-Lactamase-Producing Enterobacter and a Review of Cases in the United States From January 2009 to September 2022

**DOI:** 10.7759/cureus.60200

**Published:** 2024-05-13

**Authors:** Divya Chandramohan, Erica L Beck, Delvina Ford, Teri Hopkins, Steven D Dallas, Elizabeth Walter, Jose Cadena

**Affiliations:** 1 Infectious Diseases, University of Texas Health Science Center at San Antonio, San Antonio, USA; 2 Infection Prevention, South Texas Health System, San Antonio, USA; 3 Pharmacy, South Texas Health System, San Antonio, USA; 4 Microbiology, University of Texas Health Science Center at San Antonio, San Antonio, USA; 5 Infectious Diseases, South Texas Health System, San Antonio, USA

**Keywords:** new delhi metallo-ß lactamases (ndm), united states of america (usa), enterobacter cloacae, carbapenamase, antimicrobial resistance

## Abstract

Antimicrobial resistance is a growing problem. Novel resistance mechanisms continue to emerge, and the pipeline of antimicrobial development struggles to keep up. Antimicrobial stewardship and proper infection control are key in preventing the spread of these infections. A case of a carbapenem-resistant *Enterobacter cloacae *complex urinary isolate was identified in an 81-year-old male patient at the San Antonio Veterans Affairs hospital, Texas, USA. The patient was placed on isolation, and further testing of the isolate to other antibiotics requested. The purpose of this study is to analyze the details of reports of such cases and to review at-risk populations and appropriate treatment for resistant organisms.

## Introduction

New Delhi Metallo-ß-lactamase (NDM) producers are known to be resistant to multiple antimicrobial drug classes, thus making treatment difficult [1]. Infection control measures and antimicrobial stewardship (ASP) interventions have important roles to prevent spread in NDM [1]. Detection of these organisms initially occurred in individuals with travel to endemic locations where carbapenemases with the blaNDM gene were prevalent [2]. Plasmid-mediated transfer of these genes eventually resulted in spread across Enterobacterales, and the returning traveler is known to carry these isolates, resulting in imported infections in various parts of the world [2]. We report a case of the NDM isolate in an individual with no prior travel outside of San Antonio, Texas, USA, without any identifiable risk factors. We also perform a review of all cases reported in the USA from January 2009 to September 2022 to determine baseline characteristics, risks, and treatment.

## Case presentation

A 81-year-old male, with no relevant travel history, not of Southeast Asian origin, and with multiple comorbidities, including coronary artery disease, pulmonary fibrosis, prior stroke, obstructive sleep apnea, type 2 diabetes, and dyslipidemia, was hospitalized at the Veterans Affairs at San Antonio, Texas, USA, in 2021. Prior to this hospitalization, he was not on any antibiotics in the past year, nor did he have a history of multi-drug resistant organisms. He had a 5 mm left distal ureteral calculus and was not a candidate for general anesthesia given his cardiac risk. Therefore, he previously had a percutaneous nephrostomy tube placed two months prior to admission. After cardiac clearance for surgery, he had a pre-operative urine culture performed prior to planned cystoscopy, ureteroscopy, laser lithotripsy, and ureteral stent placement. The urine culture grew carbapenem-resistant *Enterobacter cloacae *complex and *Enterococcus faecalis*. The *E. cloacae* complex was a carbapenemase producer, detected by the Cepheid Carba-R, carbapenem resistance molecular test, and the antibiogram indicated resistance to all beta-lactams and carbapenems, sensitivity to tigecycline, and intermediate sensitivity to tobramycin. Cefiderocol susceptibilities were requested thereafter, and the isolate noted to be susceptible. Of the genotypes tested, blaNDM was detected. blaIMP, blaVIM, blaOXA-48, and blaKPC were not detected in this isolate. The patient was admitted five days later for the urologic procedure and was not initially placed on contact precautions. Contact precautions were initiated within 24 hours of his admission. The in-patient unit and the operating room (OR) staff were notified, and the case reported to Texas Department of State Health Services. The OR was terminally cleaned, and the patient was educated on precautions by the Infection Prevention and Control team. There were no secondary cases infected from this patient. In terms of treatment, the patient was empirically treated for his urine cultures prior to susceptibilities, with levofloxacin. Since the urologic procedure had already been performed when susceptibilities (Table 1) were reviewed, and this case was asymptomatic bacteriuria rather than a symptomatic urinary tract infection, treatment with other broader antibiotics was not pursued. He returned to urology follow-up a month later with no complaints.

**Table 1:** Antibiotic susceptibility profile of NDM-producing Enterobacter cloacae complex of a case patient, with interpretation of breakpoints per the CLSI, 31st edition CLSI: Clinical and Laboratory Standards Institute, MIC: Minimum Inhibitory Concentration

Antibiotic	MIC (µg/mL)	Interpretation
Amikacin	<=4	Susceptible
Aztreonam	<=2	Susceptible
Cefepime	>16	Resistant
Cefiderocol	0.12	Susceptible
Cefotaxime	>32	Resistant
Ciprofloxacin	>2	Resistant
Colistin	<0.25	Susceptible
Doxycycline	4	Susceptible
Ertapenem	>4	Resistant
Gentamicin	2	Susceptible
Imipenem	8	Resistant
Meropenem	>8	Resistant
Minocycline	4	Susceptible
Piperacillin/Tazobactam	>64	Resistant
Polymyxin B	<=0.25	Susceptible
Ticarcillin/Clavulanic acid	>128	Resistant
Tigecycline	0.5	Susceptible
Tobramycin	8	Intermediate
Trimethoprim/Sulfamethoxazole	>4	Resistant

We conducted a review of NDM cases in the literature from January 2009 to September 2022, and we have summarized individual characteristics of cases identified in the United States. Google Scholar and PubMed were search engines used to search for articles, and the keywords, "New-Delhi Metallo-ß-Lactamase", "NDM", "United States", "USA", "carbapenem resistant", "multi-drug resistant", and "CRE" were used to locate articles pertaining to this review. Duplicates of cases summarized in other articles as part of another author's literature review process were encountered. These were carefully identified and eliminated. To our knowledge, this is a complete review of all cases reported in the United States within this timeframe.

## Discussion

The study of carbapenemase-producing strains and knowledge of their epidemiological distribution has tremendous clinical significance. As we combat various bacterial strains with antibiotics, there is a slow growth in innumerous resistance mechanisms that bacteria exhibit to circumvent the deleterious effects of antibiotics [1]. Identification of these resistant isolates early is crucial to institute contact precautions and prevent further spread [2]. We present one of the few cases of NDM in *E. cloacae* complex.

A review of NDM cases in the literature from January 2009 to September 2022 showed that exposure risks included travel to endemic places, as well as acquisition through inadequately sterilized equipments. Individuals with prior travel to these countries, should prompt early consideration of NDM presence, in the setting of clinical non-improvement while on standard broad-spectrum therapy. As of January 2013, the Center for Disease Control and Prevention (CDC) reported 69 patients in the United States with NDM strains, out of which 44 were from Northeastern Illinois. Twenty-three of these cases were noted to be acquired through an exposure involving culture-positive endoscope during endoscopic retrograde cholangiopancreatography (ERCP). Subsequent to this outbreak, successful gas sterilization with ethylene oxide was achieved [3]. A retrospective cohort details a total of eight patients identified on review of cases from January 2012 to October 2012 in Colorado [4]. Following these CDC reports, there have been additional cases, with one such report arising from University of Texas MD Anderson Cancer Center at Houston in 2015. All the Enterobacterales isolates that were resistant to meropenem in this hospital were tested for susceptibility to CAZ-AVI via the E test technique, or disk diffusion, confirmed by the broth microdilution method. PCR testing of various genetic sequences expressed in CREs was undertaken. Surprisingly high rates of six out of the 11 isolates with CREs were identified as NDM, out of which four had a history of foreign travel within the previous year [5].

Of the 85 patients reviewed in Tables 2-3, *E. coli* predominated as the cause, with 53 cases, or 62.4% of total infections due to this organism. With the available data, it can be ascertained that upwards of 14 cases (16.5%) were secondary to *Klebsiella*, and nine cases (10.6%) secondary to blaNDM-1 carrying *Enterobacter*. *Enterobacter *spp., as is seen in our patient, is rarely seen as a causative organism, of which *E. cloacae* complex was implicated in eight total infections in the Unites States, nine including our case patient. Infections have occurred in all (reported) age groups, varying from 13 months to 81 years, with 14 out of 23 (60.8%) cases known to occur in older individuals over 50 years of age, likely since there is a correlation between long exposure to antibiotics, resulting in increasing resistant mutations. Our patient who carried an autochthonous isolate is stipulated to have developed genetic mutations, resulting in NDM resistance due to prior exposure, since he did not have any prior travel history. Cluster of cases are known to occur with secondary infection from an index case, the largest of which is the Illinois outbreak with infection from duodenoscopes [3]. Of the dozen cases that reported all resistant genes identified, six of the isolates possessed blaCTX-M and blaTEM-1; five isolates possessed blaOXA [5-10]. Infections are identified in the USA starting from January 2011 [11]. 

**Table 2:** Summary of cases with NDM isolated in the United States (Jan 2009-Sept 2022) secondary to species other than Enterobacter ERCP: Endoscopic Retrograde Cholangiopancreatography; NR: Not Reported; KB: Kirby-Bauer Disk Diffusion Testing; MIC: Minimum Inhibitory Concentration; TMP-SMX: Trimethoprim-Sulfamethoxazole; CAZ-AVI: Ceftazidime-Avibactam

Report	Number of cases reported	Organism	Risk factors	Genotypes reported	Patient age/gender	Isolation date	Isolation body site	State	Sensitivity profile (MIC, E test reported as µg/ml)	Treatment	Outcome
Frias et al. [3]	44	Escherichia coli	Prior ERCP	bla_NDM_	NR	Jan to Dec 2013	Clinical culture unclassified; rectal surveillance culture	Illinois	NR	NR	NR
Epson et al. [4]	8	Unknown case number of *Klebsiella pneumoniae*; *Enterobacter*; *Citrobacter*	NR	bla_NDM_	NR	Jan-Oct 2012	Urine; respiratory; rectal surveillance; other unclassified	Colorado	NR	NR	NR
Pecora et al. [6]	4	Escherichia coli	Travel to India	bla_NDM-5_, bla_CTX-M-15_, bla_TEM-1_, bla_OXA-1_	NR	2015	Urine	Massachusetts	Tigecycline (Etest 0.25); intermediate to chloramphenicol (KB); colistin (MIC 0.06)	NR	NR
Pecora et al. [6]	As above	Escherichia coli	Travel to China	bla_NDM-5_, bla_TEM-1_	NR	2015	Abscess	Massachusetts	Tigecycline (Etest 0.75); colistin (MIC 0.06)	NR	NR
Pecora et al. [6]	As above	Escherichia coli	No known risk factors	bla_NDM-1_, bla_CTX-M-15_,bla_TEM-1b_, bla_OXA-1_, bla_CMY-6_	NR	2016	Urine	Massachusetts	Tigecycline (Etest 0.125); sensitive to tetracycline (MIC 2); colistin (MIC 0.12)	NR	NR
Pecora et al. [6]	As above	Escherichia coli	Travel to India	bla_NDM-5_, bla_CTX-M-15_,bla_TEM-1_, bla_OXA-1_	NR	2016	Blood	Massachusetts	Tigecycline (Etest 0.38); sensitive to tetracycline (KB); intermediate to chloramphenicol (KB); colistin (MIC 0.12)	As above	As above
Mediavilla et al. [10]	1	Escherichia coli	Travel to India	bla_NDM-5_, bla_OXA _1,mcr1, strA, strB, aac(6’)-Ib-cr, catB3, floR, arr-3,sul1, sul2, tet(A)	76/M	Aug 2014	Urine	New Jersey	Sensitive to gentamicin, TMP-SMX	NR	NR
Green et al. [12]	2 in case patient	Escherichia coli	Travel to India	bla_NDM-1_	7/F	Nov 2012	Urine	California	Susceptible to tigecycline, fosfomycin Susceptible to fosfomycin, intermediate to tigecycline	5 days of tigecycline, then a single dose of fosfomycin	Cure
Green et al. [12]	As above	Klebsiella pneumoniae	As above	bla_NDM-1_	As above	Dec 2012	As above	As above	NR	6 days of tigecycline, concomitant with single dose of fosfomycin on day 1	Cure
Lee et al. [9]	2 in case patient	Klebsiella pneumoniae	Travel to India	bla_NDM-7_, bla_CTX-M-15_, rmtF	69/M	2012	Intra-abdominal abscess	Minnesota	Tigecycline (MIC 1); colistin (MIC <=0.25); polymyxin B (MIC <=0.25)	NR	NR
Lee et al. [9]	As above	Escherichia coli	As above	bla_NDM-7_, bla_CTX-M-15_	As above	As above	As above	As above	Tigecycline (MIC <=0.25); colistin (MIC <=0.25); polymyxin B (MIC <=0.25)	NR	NR
Aitken et al. [5]	5	Escherichia coli	Travel to Venezuela	bla_NDM-1_	22/M	2015	Scrotal abscess	Texas	NR	Tigecycline+ aztreonam+ meropenem+ colistin	Cure
Aitken et al. [5]	As above	Escherichia coli	Travel to China	bla_NDM-9_	31/M	2015	Stool	Texas	NR	Aztreonam+CAZ-AVI+tigecycline+TMP-SMX	Death
Aitken et al. [5]	As above	Klebsiella pneumoniae	Travel to Mexico	bla_NDM-1_	27/M	2015	Sputum	Texas	NR	Imipenem+ cilastatin+ ertapenem+ TMP-SMX+ tigecycline+ colistin+ amikacin	Death
Aitken et al. [5]	As above	Klebsiella pneumoniae	Travel to India	bla_NDM-1_	68/F	2015	Urine	Texas	NR	Colistin	Cure
Aitken et al. [5]	As above	Klebsiella oxytoca	No known risk factors	bla_NDM-1_	3/F	2015	Stool	Texas	NR	Colistin+ tigecycline+ cefepime	Death
Mochon et al. [13]	2 in case patient	Klebsiella pneumoniae	Travel to Pakistan	bla_NDM-1_	13 mo/M	NR	Nasal wash	California	Colistin (MIC 0.25); sensitive to tigecycline (MIC 1); minocycline (MIC 4)	Colistin	Cure
Mochon [13]	As above	Klebsiella pneumoniae	As above	bla_NDM-1_	As above	As above	Sputum	As above	Sensitive to tigecycline (MIC <=0.5); minocycline (MIC <=4)	As above	As above
Li et al. [7]	1	Klebsiella pneumoniae	Travel to Iran	bla_NDM-1_, bla_CTX-M-15_,bla_SHV-12_, bla_TEM-1_, rmtC, armA	72/F	Feb 2014	Hip wound culture	Florida	Susceptible to tigecycline, colistin	NR	NR
Mittal et al. [14]	1	Klebsiella pneumoniae	Travel to Bangladesh	bla_NDM-_1,bla_OXA-48_	42/M	NR	Elbow wound culture	New York	Intermediate to tigecycline (MIC 3); polymyxin B (MIC 0.05); synergy to CAZ-AVI, aztreonam (FIC index 0.11)	Meropenem+ tigecycline on day 1, day 2, then meropenem+ polymixin B day 3, day 4, then polymyxin B day 5 to 15, then CAZ-AVI+ aztreonam days 16 to 21, then polymyxin B on days 22 to 29, then CAZ-AVI+ aztreonam until day 62	Cure
Hardy et al. [2]	2	Klebsiella pneumoniae	Travel to Vietnam	bla_NDM_	NR	Feb; Mar 2012	Urine	Rhode island	Susceptible to tigecycline (MIC 2), colistin, polymyxin B (MIC 1) NR	NR	NR
Hardy et al. [2]	As above	Klebsiella pneumoniae	exposure to index case	bla_NDM_	NR	Mar 2012	Rectal swab	Rhode island	As above	NR	NR
Chen et al. [15]	1	Klebsiella pneumoniae	Travel to India	bla_NDM_	70/F	Aug 2016	Wound culture	Nevada	Tigecycline intermediate; fosfomycin (Etest 16)	NR	Death
Toomer et al. [16]	1	Klebsiella pneumoniae	No known risk factors	bla_NDM_	53/M	NR	Blood	Florida	Sensitive to tetracycline, tigecycline; synergy to CAZ-AVI, aztreonam (FIC 0.375)	Tigecycline+ CAZ-AVI+ aztreonam from day 1 to 8, then CAZ-AVI+ aztreonam, unknown duration	NR
Savard et al. [11]	2 in case patient	Klebsiella pneumoniae	Travel to India	bla_NDM_	60/M	Jan 2011	Sputum	Maryland	Sensitive to colistin (MIC 0.12)	NR	NR
Savard et al. [11]	As above	*Salmonella enterica* *subspecies enterica serovar* *Seftenberg*	As above	bla_NDM-1_	As above	As above	Perirectal culture	As above	Susceptible to tetracycline, tigecycline, TMP-SMX	NR	NR

**Table 3:** Summary of case reports of NDM cases in the United States (Jan 2009-Sept 2022) due to Enterobacter spp. KB: Kirby-Bauer Disk Diffusion Testing; MIC: Minimum Inhibitory Concentration; TMP-SMX: Trimethoprim-Sulfamethoxazole; CAZ-AVI: Ceftazidime-Avibactam; FIC: Fractional Inhibitory Concentration Index

Report	Number of cases reported	Organism	Risk factors	Genotype	Patient age/gender	Isolation date	Isolation body site	State	Sensitivity profile (MIC, Etest reported as µg/ml)	Treatment	Outcome
Pecora et al. [6]	1	*Enterobacter cloacae* complex	No known risk factors	bla_NDM-1_, bla_SHV-7_, bla_SHV-12_,bla_TEM-1_, bla_ACT25_	NR	2015	Blood	Massachusetts	Tigecycline (Etest 0.75); sensitive to tetracycline (MIC 4); colistin (MIC 0.06)	NR	NR
Aitken et al. [5]	1	Enterobacter cloacae	Travel to Mexico	bla_NDM-1, _bla_KPC-17_	27/M	2015	Respiratory	Texas	NR	Imipenem+ cilastatin+ ertapenem+ TMP-SMX+ tigecycline+ colistin+ amikacin	Death
Yasmin et al. [8]	1	Enterobacter hormaechei subsp.hoffmannii strain Eh	NR	bla_NDM_, bla_KPC_	4/M	NR	Blood, perianal swab	Ohio	Synergy to CAZ-AVI, aztreonam	CAZ-AVI+ aztreonam for 14 days	Cure
Siddamreddy et al. [17]	1	Enterobacter cloacae	NR	bla_NDM-1_	75/F	NR	Leg wound	Not reported	Sensitive to amikacin (MIC <=8), aztreonam (MIC <=4), gentamicin (MIC <=1), tigecycline (MIC <=1), tobramycin, intermediate to ciprofloxacin (MIC 0.5), levofloxacin (MIC 1)	Tigecycline	Cure
Nori [18]	5	Enterobacter cloacae	No known risk factors	bla_NDM_	68/F	Apr 2020	Blood, respiratory	New York	Sensitive to Colistin (MIC <=0.25); gentamicin (MIC <=2); tigecycline (MIC <=1)	Tigecycline+CAZ-AVI+aztreonam	NR
Nori et al. [18]	As above	Enterobacter cloacae	No known risk factors	bla_NDM_	57/M	Apr 2020	Urine, respiratory	New York	Sensitive to Colistin (MIC 0.5); gentamicin (MIC <=2); tigecycline (MIC <=1)	Tigecycline	NR
Nori et al. [18]	As above	Enterobacter cloacae	No known risk factors	bla_NDM_	63/M	Apr 2020	Blood, respiratory	New York	Sensitive to colistin (MIC 0.5); gentamicin (MIC 4); tigecycline (MIC <=1)	Tigecycline+ gentamicin	NR
Nori et al. [18]	As above	Enterobacter cloacae	No known risk factors	bla_NDM_	63/F	Apr 2020	Urine, respiratory	New York	Intermediate to colistin (MIC <=0.25); sensitive to gentamicin (MIC <=2); tigecycline (MIC <=1)	CAZ-AVI+ aztreonam	NR
Nori et al. [18]	As above	Enterobacter cloacae	No known risk factors	bla_NDM_	54/M	May 2020	Respiratory	New York	Intermediate to colistin (MIC <=0.25); sensitive to gentamicin (MIC <=2); tigecycline (MIC <=1)	Tigecycline,gentamicin,CAZ-AVI,aztreonam	NR
This case	1	Enterobacter cloacae	No known risk factors	bla_NDM_	81/M	Apr 2021	Urine	Texas	Sensitive to tigecycline, cefiderocol and intermediate to tobramycin	None	Clinically no concern.

Of the *E. coli* isolates with reported sensitivities, six out of seven (85.7%) was sensitive to tigecycline, followed by three out of seven (42.9%) sensitive to colisin, one each sensitive to trimethoprim-sulfamethoxazole (TMP-SMX), tetracyclines, polymyxin B, and fosfomycin. Of the *K. pneumoniae* isolates with reported sensitivities, five out of 10 (50%) showed sensitivity each to tigecycline and colistin; two isolates (20%) showed sensitivity to fosfomycin, minocycline, and polymyxin B; and one isolate was sensitive to tetracycline. Of the *E. cloacae* complex reported, all isolates, including our patient, were sensitive to tigecycline; five out of eight isolates (62.5%) were sensitive to gentamicin; four isolates (50%) to colistin; and one isolate to tetracycline and tobramycin. Our case patient had a sensitive isolate to cefiderocol, although it is uncertain whether the other reported isolates were tested to this antibiotic. This highlights empiric use of tigecycline, colistin, and gentamicin for blaNDM carrying isolates, with additional sensitivities to be requested to cefiderocol. With higher minimum inhibitory concentration (MIC) to CAZ-AVI seen in isolates, and clinical decline in several cases, the fractional inhibitory concentration (FIC) index becomes necessary. Out of the cases reviewed, only three noted synergy by this testing method. Validation of this test will be helpful in the care of patients.

Plasmid-transfer of the NDM gene from other Enterobacterales likely resulted in *Enterobacter *spp. acquiring this resistance pattern. The first ever case of *Enterobacter* with blaNDM was seen in 2015 [14]. In our case, there was no secondary infection in the seven month follow-up period after terminal disinfection. CAZ-AVI and aztreonam, tigecycline, and gentamicin have been used successfully to eradicate the infection [8,12,17]. More studies are required to assess difference in clinical outcome, eradication of the isolate via infection control practices, and different susceptibilities to antimicrobials in *Enterobacter *spp. compared to other Enterobacterales. Caution should be undertaken when reviewing clinical data from individuals with history of extensive antibiotic exposure, as well as resistance prevalent countries, with low threshold in suspecting NDM infections. To accurately identify every single case, it is also prudent for Infection control in a hospital to perform routine checks on all patients, as individuals like our patient could rarely present with no risk factors. System errors that occurred in our case can be curbed with alerts indicating buzzwords such as “highly resistant”, “isolation mandated immediately”, and “New-Delhi metallo-b-lactamase”, with explanation requested in case the provider defers isolation. 

In our review, we identified that *K. pneumoniae *and *E. coli* were predominantly implicated in carrying the NDM gene in the United States. Exposure risks to acquiring an NDM isolate are travel to endemic places and the use of inadequately sterilized equipments. Cluster of cases have been reported with secondary infection from an index case. Of the 85 patients we reviewed carrying an NDM isolate in the United States, *E. coli* predominated as the cause, with 53 cases, or 62.4% of total infections due to this organism. Meanwhile, 14 cases (16.5%) were secondary to *Klebsiella*, and nine cases (10.6%) secondary to blaNDM-1 carrying *Enterobacter*. Our case patient is the only individual case reported in the prior year in the Unites States with no relevant travel history and no immediately prior antibiotic exposure who was identified to carry a resistant isolate. Of the *E. cloacae* complex reported, all isolates, including our patient were sensitive to tigecycline; five out of eight isolates (62.5%) were sensitive to gentamicin; four isolates (50%) to colistin; and one isolate to tetracycline and tobramycin. Susceptibility testing in our case patient indicated sensitivity to cefiderocol, although this antibiotic was not tested against most of the isolates captured in this review. This highlights empiric use of tigecycline, colistin, and gentamicin for blaNDM carrying isolates, with additional sensitivities to be requested to cefiderocol.

## Conclusions

NDM-producing Enterobacterales are a growing concern with pan-resistance being reported in the past and concerns of loss of synergy to CAZ-AVI and aztreonam in the future. Worldwide spread is known to occur with multiple areas of the United States affected to date as a result of travel from a prevalent location. The plasmid-mediated transfer results in fast transmission of resistance among Enterobacterales. NDM-producing strains are being isolated in individuals with no travel history as well, given community acquisition of resistance in the recent past. This requires increased awareness for adherence to applied infection prevention practices. Overall, the importance of appropriate contact precautions, hand hygiene, antimicrobial stewardship, and rapid laboratory detection has been crucial in the prevention of highly resistant organism transmission. We must all continue to be vigilant and cautious as these resistant organisms continue to be reported. Special importance to members from prevalent countries can help with early identification, and appropriate Infection control department routine screening is necessary to identify individuals with no risk factors.
